# Sex, drugs, and arousal—two randomized trials on the effects of ketamine on sexual arousal and calcarine gyrus activity

**DOI:** 10.1177/20451253251406059

**Published:** 2026-02-12

**Authors:** Manfred Klöbl, Thomas Liebe, Gregor Dörl, Peter Stöhrmann, Clemens Schmidt, Elisa Briem, Christian Milz, Gabriel Schlosser, Maximilian Kathofer, David Gomola, Godber Mathis Godbersen, Julia Sophia Crone, Rupert Lanzenberger, Marie Spies

**Affiliations:** Department of Psychiatry and Psychotherapy, Medical University of Vienna, Vienna, Austria; Comprehensive Center for Clinical Neurosciences and Mental Health, Medical University of Vienna, Vienna, Austria; Department of Psychiatry and Psychotherapy, University of Jena, Jena, Germany; Department of Psychiatry and Psychotherapy, Medical University of Vienna, Vienna, Austria; Comprehensive Center for Clinical Neurosciences and Mental Health, Medical University of Vienna, Vienna, Austria; Department of Psychiatry and Psychotherapy, Medical University of Vienna, Vienna, Austria; Comprehensive Center for Clinical Neurosciences and Mental Health, Medical University of Vienna, Vienna, Austria; Department of Psychiatry and Psychotherapy, Medical University of Vienna, Vienna, Austria; Comprehensive Center for Clinical Neurosciences and Mental Health, Medical University of Vienna, Vienna, Austria; Department of Psychiatry and Psychotherapy, Medical University of Vienna, Vienna, Austria; Comprehensive Center for Clinical Neurosciences and Mental Health, Medical University of Vienna, Vienna, Austria; Department of Psychiatry and Psychotherapy, Medical University of Vienna, Vienna, Austria; Comprehensive Center for Clinical Neurosciences and Mental Health, Medical University of Vienna, Vienna, Austria; Department of Psychiatry and Psychotherapy, Medical University of Vienna, Vienna, Austria; Comprehensive Center for Clinical Neurosciences and Mental Health, Medical University of Vienna, Vienna, Austria; Vienna Cognitive Science Hub, University of Vienna, Vienna, Austria; Department of Psychiatry and Psychotherapy, Medical University of Vienna, Vienna, Austria; Comprehensive Center for Clinical Neurosciences and Mental Health, Medical University of Vienna, Vienna, Austria; Department of Psychiatry and Psychotherapy, Medical University of Vienna, Vienna, Austria; Comprehensive Center for Clinical Neurosciences and Mental Health, Medical University of Vienna, Vienna, Austria; Vienna Cognitive Science Hub, University of Vienna, Vienna, Austria; Department of Psychiatry and Psychotherapy, Medical University of Vienna, Waehringer Guertel 18-20, Vienna 1090, Austria; Comprehensive Center for Clinical Neurosciences and Mental Health, Medical University of Vienna, Vienna, Austria; Department of Psychiatry and Psychotherapy, Medical University of Vienna, Vienna, Austria; Comprehensive Center for Clinical Neurosciences and Mental Health, Medical University of Vienna, Vienna, Austria

**Keywords:** functional magnetic resonance imaging, ketamine, resting-state, sex differences, sexual arousal

## Abstract

**Background:**

Ketamine, a well-established antidepressant and dissociative anesthetic, is also used recreationally in the club and chemsex scene. Survey and qualitative data suggest that while ketamine facilitates chemsex encounters, it diminishes the intensity of the sexual experience.

**Objectives:**

To investigate this phenomenon from a neuroscientific perspective while considering ketamine’s sex-specific effects.

**Design:**

Two randomized, placebo-controlled crossover studies using intranasal S-ketamine (double-blinded) or intravenous racemic ketamine (single-blinded).

**Methods:**

Subjective sexual arousal in response to a newly compiled set of erotic stimuli was assessed following subacute S-ketamine and late racemic ketamine administration across two studies. Overall, 67 healthy volunteers (26 females) participated in the studies. Functional magnetic resonance imaging (fMRI) was performed during sexual arousal assessment under late racemic ketamine exposure, with both studies also incorporating resting-state fMRI assessments.

**Results:**

Subacute S-ketamine reduced sexual arousal to heterosexual stimuli in women (β = −0.21, CI_95_ = (−0.36, −0.06)) and, to a lesser extent, to lesbian stimuli in men (β = −0.16, CI_95_ = (0.003, −0.33)). It also diminished sexual aversion to gay stimuli in both sexes (β ⩾ 0.18, CI_95_ ⩾ (0.03, 0.32)). Conversely, late racemic ketamine decreased sexual arousal to heterosexual stimuli in men (β = −0.17, CI_95_ = (−0.31, −0.02)) while exacerbating sexual aversion to gay stimuli in women (β = −0.24, CI_95_=(−0.36,−0.12)). Furthermore, late ketamine administration resulted in reduced calcarine gyrus activation in men compared to women, independent of sexual arousal (β ⩽ −0.23, CI_95_ ⩽ (−0.52, 0.05)). This finding was confirmed for resting activity under subacute ketamine (β = −0.18, CI_95_ = (−0.32, −0.04)).

**Conclusion:**

Our results align with reports of diminished sexual arousal under ketamine, while the reduced sexual aversion may play a role in facilitating chemsex. The heightened sexual aversion in women and the distinct calcarine gyrus activity modulation may relate to previously documented sex-dependent ketamine effects on stress resilience and psychosis-like symptoms.

**Trial registration:**

Both studies were registered at clinicaltrials.gov: NCT05320120 (2022-04-08), NCT05320107 (2022-04-08).

## Introduction

Initially developed as an anesthetic, ketamine has evolved into a versatile drug in both clinical practice and research.^1^ Beyond its medical applications, ketamine is highly utilized in the chemsex^2–4^ and club scene.^5–7^ In particular, among men who have sex with men, drugs are consumed to alter or intensify the sexual and emotional experience.^3^ However, survey data indicate that ketamine is more frequently reported to worsen sexual experiences than to enhance them^8^ and anecdotal evidence suggests its use to facilitate passive anal intercourse.^9^ Still, the underlying effects of ketamine on sexual arousal remain unexplored from both neural and behavioral perspectives.

Even at subanesthetic doses, Ketamine induces changes in neural activity and functional connectivity, as demonstrated through functional magnetic resonance imaging (fMRI) and other modalities. These effects are particularly pronounced in visual regions and have been linked to reduced attention and vigilance, as well as psychotomimetic effects.^10–16^ The primary visual region anatomically corresponds to the calcarine cortex, which contains a representation of the retinotopic map.^17^ Beyond its role in the low-level processing of, for example, saccades and spatial attention,^18,19^ the calcarine cortex is also activated by mental imagery.^20^ Studies have shown that ketamine reduces functional connectivity between visual brain regions,^13^ reduces visual cortex activation during smooth pursuit eye movements,^14^ and reduces cerebral blood flow in the primary visual cortex of individuals with schizophrenia.^16^

Notably, a prohedonic component has been proposed in connection with ketamine’s antidepressant properties,^21,22^ suggesting its potential to enhance reward-seeking behavior. Preclinical and clinical evidence further indicate that ketamine’s rewarding effects are context-dependent, with higher doses more frequently consumed in unfamiliar environments (e.g., clubs and parties).^23^ These findings could provide starting points for a mechanistic and behavioral understanding of recreational ketamine use in stimulating, pleasure-oriented environments, such as the chemsex scene.

When examining ketamine’s effects on sexual arousal, it is crucial to consider sex differences for both sexual arousal and ketamine’s effects. Furthermore, sexual arousal has been shown to be influenced by gender identity, in addition to the type of stimuli and the method of assessment.^24^ Animal studies have revealed potential sex differences in depression-related behavioral responses to ketamine,^25–28^ while human studies have linked sex differences in ketamine’s antidepressant effects to sex hormones.^29^ The predominance of men who have sex with men in the chemsex scene^30,31^ underscores the importance of sex and gender as key factors, though studies from France and Italy highlight significant chemsex participation among women as well.^8,32^

In this context, we hypothesized that ketamine’s effects on sexual arousal and aversion, as well as brain activation, could vary in two ways: If ketamine exerts prohedonic effects also in healthy individuals, we would expect increased sexual arousal to stimuli that were rated as arousing under placebo. Conversely, decreased sexual arousal would corroborate reports of worsened sexual experiences under ketamine. Given known differences in sexual preferences, these effects would necessarily be modulated by the sex of the participants and the sexual orientation of the stimuli.^24,33^ These hypotheses were tested in healthy volunteers across two studies examining subacute and late ketamine effects (relative to the peak of the subjective high) using self-reported sexual arousal and fMRI data collected at rest and during a sexual arousal task. Since we only enrolled healthy individuals, benefits and harms were limited to the expense allowance and potential drug side effects, respectively.

## Methods and materials

We investigated the effects of ketamine on sexual arousal in two studies using a newly compiled set of erotic stimuli. Participants were recruited through flyers posted on designated message boards at the General Hospital of Vienna and throughout the city. Inclusion criteria for both studies encompassed age 18–55 years, general physical and mental health based on medical history, physical examination, and Structured Clinical Interview for the Diagnostic and Statistical Manual of Mental Disorders (DSM; SCID), right-handedness to ensure equal lateralization, no past or present substance abuse, and no lifetime use of ketamine. Studies 1 and 2 used the SCID for DSM-IV^34^ and DSM-5,^35^ respectively. Both versions represent standards in psychiatric diagnostics and have been validated extensively (e.g., Osório et al.,^36^ Gorgens et al.^37^). Participants were excluded if they had a current or past history of psychiatric or neurological disorders, current illnesses requiring treatment, current or past substance abuse, were pregnant or breastfeeding, or had a contraindication to ketamine or MRI investigations. In addition, Study 2 did not permit any lifetime use of ketamine. After passing the initial screening and providing written informed consent, participants were enrolled by the study physician or medical students under supervision. Dropouts based on overall study objectives were replaced, and recruitment ended after the intended sample sizes were achieved.

Randomization, as described below, was performed by a team member not further involved in the studies. The randomization lists were stored on a password-protected network drive and were only sent to the pharmacy of the Medical University of Vienna for drug preparation upon participant enrollment.

All study procedures were performed at the Medical University of Vienna. fMRI data were acquired using a Siemens Prisma 3T scanner at the university’s High-field Magnetic Resonance Center. The analyses in this manuscript were exploratory, and the measures were not registered as primary or secondary outcomes. Descriptive statistics are provided as median ± interquartile range. The reporting of this study conforms to the CONSORT guidelines.^38^ For details on participant inclusion and exclusion, see the accompanying CONSORT flow diagrams.

### Compiling the novel stimulation material

Following recent recommendations advocating for women to select visual erotic stimuli in sexual arousal research,^39^ we developed a novel set of stimulation materials. Research staff and students involved in the studies were tasked with selecting heterosexual, female (lesbian), and male (gay) homosexual images from a public database of pornographic movie stills. Alternative sources were permitted if no suitable images were identified. To ensure anonymity, participants labeled the images with their self-identified gender (male, female), sexual orientation (heterosexual, bisexual, homosexual), and a unique random code generated once per individual. This process maintained confidentiality while indicating which images were chosen by the same person.

In addition to meeting image quality standards, the selected stimuli had to fulfill the following criteria: depict exactly two individuals engaged in intercourse, exclude fetishes to ensure broad appeal, and elicit subjective sexual arousal when the scene’s orientation aligned with that of the selector However, we must note that, apart from these criteria, no validation and reliability assessments have been conducted with the stimuli to date. From the compiled images, 36 of each orientation (108 total) were chosen, prioritizing those selected by individuals whose self-reported gender and orientation matched the depicted scene (e.g., lesbian stimuli preferred images chosen by lesbian or bisexual women). If fewer than 36 images met these criteria, a balanced selection was made, considering all other variables (i.e., selector identity, gender, and orientation).

### Study 1—Reported sexual arousal after intranasal S-ketamine

Study 1 investigated the subacute ketamine effects on subjective sexual and general arousal as well as on resting fMRI brain activity.

#### Study procedure and design

The sample size was estimated based on a calculated effect size of *f* = 0.25 (*r* = 0.60, 1 – β = 0.80).^40,41^ Reliability was assumed to be *r* = 0.54.^42^ A within-subject analysis of variance (ANOVA) and two covariates yielded a sample size of *N* = 33. Participants were recruited from the beginning of June 2022 to the end of April 2023 and followed up until mid-June 2023. The first subject was enrolled on June 9, 2022. Thirty participants (24.0 ± 2.0 years, 15 females) received 56 mg intranasal S-ketamine (Spravato) or placebo (saline) in a randomized, double-blind, crossover fashion with 14 ± 14.5 days between sessions. This drug application scheme was chosen to maximize comparability with the clinical practice for the antidepressant use of S-ketamine. The order of conditions was block-randomized with a block size of six. Blinding was achieved by using identical nasal spray bottles and covering the labels with opaque adhesive tape.

#### Sexual and general arousal task

Participants rated all 108 images from our stimulus set, presented in random order on a laptop, using visual analog scales in the rooms of the Department of Psychiatry and Psychotherapy of the Medical University of Vienna. Sexual arousal ratings ranged from “strongly turning off” to “strongly turning on,” whereas general arousal ratings ranged from “very calming” to “very exciting.” The task commenced 146 ± 14 min (approximately 2.5 h) after drug administration (the acute phase was reserved for MRI scans presented elsewhere).

#### fMRI acquisition, processing, and modeling

Resting-state fMRI data were acquired 45 ± 4 min after drug administration over an 8-minute period using the following parameters: echo time/repetition time (TE/TR) = 30/1114 ms, 2-mm isotropic resolution, a field of view of 208 × 208 × 144 mm, multiband factor 4, generalized autocalibrating partial parallel acquisition (GRAPPA) factor 2, and a bandwidth of 2290 Hz/Px.

Unless otherwise specified, preprocessing was conducted in Statistical Parametric Mapping 12 (SPM12). The steps included physiological noise modeling and regression,^43^ slice-wise motion correction,^44^ wavelet despiking,^45^ slice-timing correction, distortion correction,^46^ realignment across sessions, and normalization (preserving the original voxel dimensions)^47^ via a T1-weighted scan. These steps were followed by gray matter masking and Gaussian smoothing with a kernel size equivalent to three times the voxel dimension. Nuisance regression anatomical CompCor^48^ and bandpass filtering^49^ (0.01–0.10 Hz). Surrogates of resting brain activity were computed as percent amplitude of fluctuation (PerAF^50^) from smoothed data and regional homogeneity (ReHo^51^) from unsmoothed data.

#### Statistical analysis

Bayesian inference on the level of single-image ratings was conducted using mixed effects models in R using Integrated Nested Laplace Approximation (INLA^52^). This allowed for the inclusion of partially available data without special handling of missing values. Model selection via the Watanabe–Akaike Information Criterion resulted in the following specification:

$$\begin{array}{l} \mathrm{SA}\sim \mathrm{drug}*\mathrm{orient}*\mathrm{sex}*\mathrm{time}+\mathrm{age}+\mathrm{habit}+\\ \mathrm{order}+\mathrm{GA}*\mathrm{sex}*\mathrm{orient}+\left(1|subject\right)+(1|\mathrm{stim}) \end{array}$$

Sexual arousal (*SA*) scores were scaled to the interval (0, 1) and modeled with a beta distribution using a logit link function. Given that chromosomal sex influences ketamine’s effects^29^ and reported SA,^24^ the model includes an interaction between drug, participant *sex*, and stimulus *orient*ation, adjusted for the logarithm of the *time* since drug application. Additional covariates comprised participant *age*, potential *habit*uation effects from repeated measurements, *order* of drug application, and Fisher-*z*-transformed general arousal (*GA*) for each stimulus orientation and participant *sex*. Crossed random effects were incorporated for *subjects* and *stim*uli.

INLA reports effects with 95% credible intervals (CIs), where intervals not overlapping zero serve as a rough equivalent to *p*-values < 0.05. This Bayesian multilevel approach further mitigates concerns regarding multiple comparisons, eliminating the need for additional corrections.^53^

### Study 2—Reported and neural sexual arousal after intravenous racemic ketamine

Study 2 complemented the investigation of subacute ketamine effects in Study 1 by brain activation and subjective responses collected via an fMRI SA task and resting fMRI brain activity during late ketamine effects.

#### Study procedure and design

The target sample size was chosen as typical for pharmaco-fMRI studies. Participants were recruited from the beginning of June 2022 until mid-June 2023 and followed up until the end of November 2023. The first subject was enrolled on August 9, 2022. Thirty-seven participants (23.7 ± 2.2 years, 21 females) received an intravenous dose of 0.5 mg/kg racemic ketamine diluted with saline or saline alone (placebo) over 40 min in a randomized, single-blind, crossover fashion. The infused solutions were indistinguishable to the participants. The sessions were 19 ± 28 days apart. This drug application scheme was chosen to maximize comparability with the majority of the currently published ketamine research in healthy individuals. The order of conditions was block-randomized with a block size of 8. To account for variations in sexual orientation, they completed the Klein Sexual Orientation Grid (KSOG) on a tablet. The KSOG is widely used in sex research and assesses past, present, and ideal sexuality. For a reprint of the publication introducing the KSOG, see Klein.^54^ For a confirmatory factor analysis highlighting some limitations of the questionnaire, see Cramer et al.^55^

#### fMRI sexual arousal task

While undergoing MRI scanning, participants viewed 54 images from our stimulus set in random order and rated their SA on a four-point Likert scale (strongly turning on, turning on, turning off, strongly turning off^24^) using an MR-compatible input device. Images were presented in 18 blocks, each containing 3 images of the same sexual orientation. Each stimulus was displayed for 7 s with baseline periods of 21 s between blocks. The task commenced 325 ± 12 min (approximately 5.5 h) after drug application.

#### fMRI acquisition, processing, and modeling

fMRI data of the SA task were acquired using the following parameters: TE/TR = 30/1600 ms, 3 mm isotropic resolution, field of view = 210 × 210 × 144 mm, multiband factor = 2, bandwidth = 1830 Hz/Px. Resting-state fMRI data were collected 249 ± 3 min (approximately 4 h) after drug administration for 10 min, with TR = 800 ms, multiband factor = 4, pixel bandwidth = 2040 Hz/Px, and otherwise identical parameters.

Preprocessing and resting-state procedures were performed as in Study 1. For the SA task, session-wise first-level models in SPM12 were constructed with one regressor per stimulus condition (heterosexual, lesbian, and gay), anatomical CompCor regressors, no high-pass filtering, and the “FAST” autoregression model.^56^

#### Statistical analysis—Responses

Subjective ratings of SA were again modeled using INLA with a beta distribution and a logit link function. Responses were scaled to the interval (0.1, 0.9) following the quantification suggested in Safron et al.^57^ (e.g., “strongly turning on” corresponds to the interval (0.8, 1.0)). The model was similar to that in Study 1, but was adjusted for the sex-specific first principal components derived from the sexual attraction, behavior, and fantasy items of the KSOG instead of GA. This adjustment accounted for variability in the responses for each orientation of the stimuli due to the sexual orientation of the participants. The final model, after model selection, was specified as:

$$\begin{array}{l} \mathrm{SA}\sim \mathrm{drug}*\mathrm{orient}*\mathrm{sex}*\mathrm{time}+\mathrm{age}+\mathrm{habit}\\ +\mathrm{order}+\left(KSOG_{f}+KSOG_{m}\right)*\mathrm{orient}\\ +\left(1|\mathrm{subject}\right)+(1|\mathrm{image}) \end{array}$$

#### Statistical analysis—fMRI activation during sexual arousal task

Based on our previous fMRI investigation of SA,^24^ the ventral striatum was first examined and did not reveal reliable effects of any predictor. Therefore, we performed a whole-brain group-level analysis using the Sandwich Estimator (SwE) toolbox version 2.2.2.^58^ The design matrix was modeled as:

$$\begin{array}{l} \mathrm{activation}\sim \mathrm{drug}*\mathrm{orient}*\mathrm{sex}+\mathrm{age}+\mathrm{habit}\\ +\mathrm{order}+\mathrm{time}+\left(KSOG_{f}+KSOG_{m}\right)*\mathrm{orient} \end{array}$$

SwE employs marginal models to account for repeated measures without random effects. “Type C2” small sample adjustment and “approx. II” degrees of freedom estimation were used, which are ideal for complete datasets. Cluster-level correction was achieved using AFNI’s 3dFWHMx followed by 3dClustSim with median autocorrelation parameters, and multiplicity was controlled with the Šidak method. The median activation from significant clusters was then extracted and further analyzed using INLA with the model:

$$\begin{array}{l} \mathrm{activation}\sim \mathrm{drug}*\mathrm{orient}*\mathrm{sex}+\mathrm{age}+\mathrm{habit}\\ +\mathrm{order}+\mathrm{time}+\left(KSOG_{f}+KSOG_{m}\right)*\\ \mathrm{orient}+\left(1|\mathrm{subject}\right) \end{array}$$

### Statistical analysis—Resting activity

Because the fMRI SA task revealed ketamine- and sex-related effects independent of stimulus orientation, we additionally examined the resting activity of the affected region in both studies for the generalizability of the finding. The following model was employed:

$$\begin{array}{l} \mathrm{activity}\sim \mathrm{drug}*\mathrm{sex}+\mathrm{age}+\mathrm{habit}+\\ \mathrm{order}+\mathrm{time}+\left(1|\mathrm{subject}\right) \end{array}$$

PerAF was modeled using a gamma distribution with a log link, while ReHo was modeled using a beta distribution with a logit link, in accordance with their natural distributions.

### Positionality statement

The studies presented in this manuscript were conducted by researchers with diverse disciplinary and personal backgrounds to examine the effects of ketamine on subjective SA in healthy individuals. A central aspect of our hypothesis was the use of ketamine in the context of chemsex, which prompted investigation of its potential influence on SA. Our intention is to contribute positively to the scientific understanding of sexuality and the effects of ketamine rather than to pathologize recreational drug use, sexual preferences, or identities. In designing the task, we sought to compile a diverse and inclusive set of erotic stimuli, while recognizing that complete representation is not achievable, particularly within the constraints of a controlled study. We acknowledge that sexuality is a sensitive and multifaceted phenomenon, and our design captures only a limited aspect of its complexity. The development of novel stimulus material was also motivated by the reliance on outdated erotic stimuli in our earlier research. While our previous studies often revealed strong effects of ketamine in healthy individuals, our goal here is to present findings objectively, with a nuanced and differentiated discussion. Despite our efforts, limitations regarding inclusivity, diversity, and the scope of a visual task remain, and we therefore present our results with caution while encouraging future research that advances sensitivity and inclusivity in this field.

## Results

**Figure 1.**
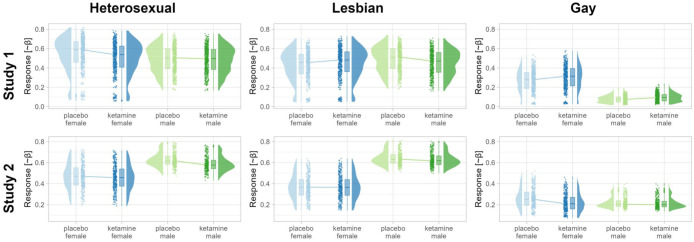
Fitted reported sexual arousal per subject and trial. The values have been adjusted for the model covariates.

**Figure 2.**
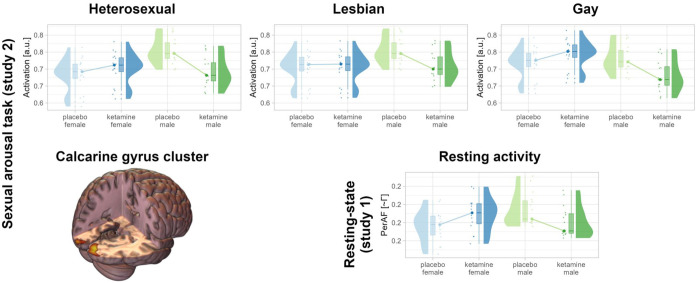
Fitted task activation and resting activity per subject. The values have been adjusted for the model covariates. The rendering shows the significantly affected cluster in the calcarine gyrus.

Table 1 provides an overview of the demographics of the participants per study. Ketamine-related effects on SA are summarized in Table 2 for the model with female sex, heterosexual stimuli, and placebo as reference conditions and depicted in Figure 1. The full model and models with different reference conditions are detailed in the Supplemental File. Results for the SA task activation and resting activity are presented in Tables 3 and 4 and visualized in Figure 2.

**Table 1.** Study demographics.

Value	Study 1	Study 2
*N* (female)	30 (15)	37 (21)
Age (median ± IQR years)	24.0 ± 2.0	23.7 ± 2.2

**Table 2.** Ketamine-related effects on reported sexual arousal and calcarine activation with 95%CI.

Effect	Study 1: Subacute S-ketamine		Study 2: Late racemic ketamine			
	Reported sexual arousal		Reported sexual arousal		Calcarine gyrus activation	
	Estimate	95% CI	Estimate	95% CI	Estimate	95% CI
Ketamine	−**0.21**	**[**−**0.36**, −**0.06]**	−0.05	[−0.16, 0.07]	0.09	[−0.10, 0.28]
Ketamine × lesbian	**0.31**	**[0.10, 0.52]**	0.04	[−0.11, 0.20]	−0.09	[−0.36, 0.18]
Ketamine × gay	**0.38**	**[0.18, 0.59]**	−**0.19**	**[**−**0.35**, −**0.03]**	0.04	[−0.23, 0.31]
Ketamine × male	0.18	[−0.05, 0.40]	−0.12	[−0.30, −0.06]	−**0.41**	**[**−**0.69**, −**0.12]**
Ketamine × lesbian × male	−**0.44**	**[**−**0.75**, −**0.14]**	0.06	[−0.19, 0.31]	0.18	[−0.23, 0.58]
Ketamine × gay × male	−0.10	[−0.40, 0.20]	**0.33**	**[0.07, 0.59]**	0.03	[−0.37, 0.43]

Female subject sex, heterosexual stimuli, and the placebo condition were used as reference categories. Effects with 95% CIs not covering 0 are highlighted in bold.

CI, credible intervals.

**Table 3.** Significant whole-brain results.

Contrast	Test	Cluster size [voxel]	*p*-Value	Corrected *p*-Value	Peak coordinates
Subject sex × stimulus orientation	*X* ^2^	94 (+30)	0.0090	0.0355	15/−88/−4 (−12/−103/−7)
(male–female) × (hetero–gay)	*z*	220	0.0005	0.0035	18/−94/−4
(male–female) × (lesbian–gay)	*z*	305	<1e-5	0.0003	−12/−103/−7

In the *X*² test, the otherwise connected cluster was split into two; the smaller part is presented in parentheses. The *p*-value refers to the larger part only. *X*²-test corrected for four interaction effects. *z*-Tests additionally corrected for three pairwise comparisons.

**Table 4.** Ketamine-related effects on calcarine gyrus resting activity with 95% CIs.

Effect	Study 1: Subacute S-ketamine				Study 2: Late racemic ketamine			
	PerAF		ReHo		PerAF		ReHo	
	Estimate	95% CI	Estimate	95% CI	Estimate	95% CI	Estimate	95% CI
ketamine	0.09	[−0.02, 0.19]	0.02	[−0.22, 0.26]	−0.04	[−0.14, 0.7]	−0.01	[−0.18, 0.16]
ketamine × male	**−0.18**	**[−0.32, −0.04]**	−0.18	[−0.51, 0.16]	−0.05	[−0.21, 0.10]	0.02	[−0.23, 0.26]

CIs, credible intervals; PerAF, percent amplitude of fluctuation; ReHo, regional homogeneity. Effects with 95% CIs not covering 0 are highlighted in bold.

### Study 1—Reported sexual arousal after intranasal S-ketamine

No response data had to be excluded. In women, ketamine decreased reported SA to heterosexual stimuli (β = −0.21, CI_95_ = (−0.36, −0.06)) while reducing aversion to gay stimuli (β = 0.18, CI_95_ = (0.03, 0.32)). In addition, ketamine elevated women’s reported SA for lesbian stimuli, but only in comparison to heterosexual stimuli (β = 0.31, CI_95_ = (0.10, 0.52)). In men, ketamine reduced aversion to gay stimuli (β = 0.25, CI_95_ = (0.09, 0.41)) but slightly decreased reported SA for lesbian stimuli (β = −0.16, CI_95_ = (0.003, −0.33)). This reduction in men’s arousal for lesbian stimuli was particularly pronounced when compared to women’s responses (β = −0.27, CI_95_ = (−0.48, −0.04)). Notably, strong correlations were observed between sexual and GA, with a marked increase in GA for men’s ratings of gay stimuli (see Supplemental File). Overall, subacute S-ketamine effects were associated with diminished SA and aversion.

### Study 2—Reported sexual arousal after intravenous racemic ketamine

The response data of one participant were excluded due to a potential individual bias, as nearly all stimuli were rated as “strongly dislike.” In addition, responses from two measurements were not recorded due to hardware errors. Consequently, the behavioral analysis included complete data from 33 participants and two single runs. For the fMRI analysis, data from two participants were excluded due to severe motion artifacts, leaving 35 datasets.

On average, female participants reported to be “mostly attracted to men” and identified as “mostly heterosexual.” Average reports for male participants showed that they were “mostly/only attracted to women” and identified as “heterosexual only.” See Supplemental File for details on the KSOG variables.

In women, compared to placebo, ketamine further increased aversion to gay stimuli (β = −0.24, CI_95_ = (−0.36, −0.12)), an effect particularly pronounced when compared to men’s responses (β = −0.21, CI_95_ = (−0.41, −0.01)). In men, ketamine decreased reported SA for heterosexual stimuli (β = −0.17, CI_95_ = (−0.31, −0.02)).

### Ketamine effects on task activation and resting brain activity

No significant ketamine-related effects on whole-brain activation during the SA task were observed. However, a participant sex by stimulus orientation interaction was identified in the visual cortex (*p* = 0.0355; see Supplemental File for baseline activations). The affected cluster primarily encompassed the calcarine gyrus, extending into the lingual and lateral occipital gyri. Detailed analysis revealed that ketamine reduced activation in this cluster in men compared to women across all stimulus conditions, with a less pronounced effect for lesbian stimuli (hetero: β = −0.41, CI_95_ = (−0.69, −0.12), lesbian: β = −0.23, CI_95_ = (−0.52, 0.05), gay: β = −0.38, CI_95_ = (−0.67, −0.10); see also Supplemental File). At the whole-brain level, this effect was evident only at relaxed thresholds but extended considerably along the lingual gyrus, reaching the calcarine, hippocampal, and parahippocampal gyri (see Supplemental File).

In the calcarine gyrus cluster, resting activity estimated via PerAF in Study 1 showed the same effect, that is, reduced activity in men compared to women following ketamine administration (β = −0.18, CI_95_ = (−0.32, −0.04)). This effect was much less reliable for ReHo (β = −0.18, CI_95_ = (−0.51, 0.16)) and was not detectable in Study 2 (Table 4).

## Discussion

In the current work, we examined the effects of ketamine versus placebo on SA across two studies utilizing a novel, specifically curated dataset of erotic stimuli. Study 1 showed that in the subacute phase, around 2.5 h after intranasal S-ketamine application, SA was attenuated for heterosexual stimuli in female participants and to a lesser degree for lesbian stimuli in male participants. Moreover, aversion to gay stimuli was attenuated for both sexes. Conversely, in Study 2, around 5.5 h after intravenous racemic ketamine application, men exhibited reduced reported SA for heterosexual stimuli, while women demonstrated increased aversion to gay stimuli. In addition, Study 2 identified a ketamine-related reduction in calcarine gyrus activation in men relative to women, irrespective of stimulus orientation. This effect was also evident in resting activity approximately 45 min, but not 4 h after ketamine administration.

### Ketamine as a sexual drug

Our finding that ketamine partially reduced ratings of stimuli rated as sexually arousing under placebo 2.5 h after administration is consistent with survey data.^8^ While the subacute reduction in SA was mainly observed for heterosexual stimuli in women and to a lesser extent for lesbian stimuli in men, in the late phase (i.e., 5.5 h after administration), we observed reduced SA to heterosexual stimuli only in men. Apart from differences in study design, such as administering S- versus racemic ketamine or using a visual analog scale versus a Likert scale, this pattern could have two causes: On the one hand, the effects of ketamine on SA may not monotonically cease over time. On the other hand, the groups in both studies differed qualitatively in their responses to the placebo. Within-gender differences in preferences for pornographic material in predominantly heterosexual individuals have also been reported across previous studies.^59–62^ It is therefore conceivable that group differences play a role. However, in both studies, ketamine reduced the average responses to stimuli that were sexually arousing under placebo, in men and women.

Besides facilitating intercourse in men that have sex with men (e.g., improving passive anal intercourse^9^—potentially also through ketamine’s analgesic effects, increasing confidence/reducing inhibition^3^), there might be further attractive properties of ketamine that do not directly increase SA: In a similar vein to ketamine, survey data reveals that 3,4-methylendioxymethamphetamine (MDMA^8,63^) is also commonly used to enhance sexual pleasure but, at least in men, impairs sexual activity.^64^ Based on its ability to enhance closeness, intimacy, and sensual perception,^65^ it has been hypothesized that MDMA mimics the post-orgasmic state through increased prolactin levels.^66^ Since ketamine also elevates serum prolactin levels^67^ and promotes social touch-seeking behavior,^68^ similar mechanisms might underlie the consumption of both substances in sexual contexts. However, consuming ketamine in the context of chemsex differs substantially from participating in a clinical study. Thus, targeted investigations are needed to generalize our results beyond the clinical study context.

In addition to attenuating sexually arousing responses, ketamine also reduced aversive responses approximately 2.5 h post-administration. Consistent with prior research, these reductions were most notable for stimuli depicting gay intercourse.^33^ The diminished SA and aversion are unlikely to be the result of a reduction in GA, as this was adjusted for in Study 1, and we actually observed an increase in GA to gay stimuli post-ketamine only in men (see Supplemental File for unadjusted models). In addition to being the result of a generally attenuated sexual experience, the reduced sexual aversion could be interpreted in the light of increased openness induced by ketamine.^69^ However, this argument should be followed with caution: While increased openness (to experiences) may be an additional facilitating factor among men who have sex with men, it should not be interpreted as increasing the willingness to engage in fundamentally aversive sexual practices.

In Study 2, 5.5 h post-administration of racemic ketamine, and contrary to Study 1, male participants did not report reduced SA to lesbian stimuli but to heterosexual ones. Interestingly, at this time, women rated gay stimuli as more aversive after ketamine than after placebo. Of note, the absence of reduced aversion would not contradict the potential facilitating use of ketamine in the chemsex scene, where acute and subacute effects are of primary interest. While these findings again highlight sex-specific effects, they also indicate that ketamine can increase aversive responses. Supporting this, a study in mice demonstrated that ketamine-induced taste aversion, potentially exacerbating existing frustration.^70^ In addition, ketamine was shown to enhance resilience to chronic stress in male but not female mice.^71^ The increased aversion observed in female participants during the late, but not subacute, effects of ketamine may reflect an accumulation of negative emotions over time, such as stress from study procedures or fatigue-induced frustration, potentially explaining this differential finding.

### Ketamine as a visual drug

The reduced activation for stimuli of all orientations, along with diminished resting activity in the calcarine gyrus observed in men compared to women, indicates that ketamine’s effects might be primarily driven by participant sex, with only a secondary influence of stimulus content. The calcarine gyrus forms part of the primary visual cortex, but has also been linked to attention and reward processing.^72,73^ This might explain, on the one hand, the interaction effect of participant sex by stimulus orientation—suggesting sex and stimulus-dependent attention and reward—and, on the other hand, the effect of ketamine across stimulus orientations.

Our findings align with prior studies in healthy male individuals that reported reduced activation in primary visual regions under ketamine during smooth pursuit eye movements and visual oddball tasks.^14,15^ Similarly, earlier research demonstrated reduced blood flow in primary visual regions at rest under ketamine in patients with schizophrenia.^16^ The visual components of ketamine’s effects have been consistently reported across various modalities, though often without accounting for participant sex (see Schwertner et al.,^74^ Frohlich et al.^75^ for reviews). These findings related to the visual cortex may reflect ketamine’s psychotomimetic effects,^13^ which is consistent with the more pronounced psychosis-like side effects observed in men.^29^ The observation that ketamine influenced resting activity at 45 min but not at 4 h post-administration, while a task-related effect was still evident after 5.5 h, could be attributed to the differing psychotomimetic properties of R-ketamine (present in the racemic mixture) compared to S-ketamine.^76^ Alternatively, the varying time scales of intrinsic (resting-state) versus stimulus-related (task) activity might explain these differences, but further investigation is warranted.

### Moderators of ketamine’s effects on sexual arousal

While our findings corroborate the evidence from survey data on ketamine’s dampening effect on sexual experiences,^8^ it should be noted that our study only reflects a small part of this complex relationship. The effects of ketamine on SA depend on several influential factors, including the sex of the participants, the sexual orientation of the stimuli, and the time between drug administration and stimulus exposure. Furthermore, since sexual orientation and gender identity influence SA,^24,33,57^ they could also modulate the effects of ketamine on sexual experiences. SA also extends far beyond the reaction to visual stimuli, and sexual experiences comprise more than arousal. Although we found no evidence of prohedonic effects of ketamine on subjective SA, such effects may be observed in patients with depression, for whom sexual dysfunction is a common symptom.^77^ Thus, targeted investigations are required to test whether ketamine also temporarily dampens SA in patients with depression or if a prohedonic effect prevails. Future studies could also examine the potential moderating effects of sexual orientation or gender identity on ketamine’s impact on SA. In addition, the temporal trajectory of the effects requires further investigation, as we only examined subacute and late stimulus exposure.

### Limitations

Although our novel stimulus material was carefully selected with the aim of maximizing inclusivity, no validation or reliability data is currently available. The sample size considerations for both studies did not include sex as a primary variable of interest. This resulted in potentially underpowered subgroups of male and female participants and an unequal sex distribution in Study 2. Thus, our results need to be replicated in a larger, sex-balanced sample. The direct comparability of the two studies is constrained by differences in drug administration protocols and necessary adaptations to the fMRI version of the SA task. In addition, due to logistical factors and task design limitations, Study 1 was adjusted for GA, whereas Study 2 accounted for participants’ sexual orientation. The use of a simplified four-point Likert scale in Study 2 for stimulus ratings may also have influenced responses compared to the visual analog scale employed in Study 1.

## Conclusion

Our findings of reduced subjective SA and aversion under subacute ketamine in both men and women support survey data on its use in the chemsex scene. Although ketamine partially diminishes the sexual experience, it is unclear whether the reduced sexual aversion plays a facilitating role in the context of chemsex. In the late phase, the sex-specific effects of ketamine became more pronounced, with men reporting reduced SA, but women actually showing increased sexual aversion. We also observed sex-specific effects on calcarine gyrus activation under ketamine, with relative increases in women and relative decreases in men. These differential findings may be related to previous reports of ketamine-induced increased stress resilience, but also stronger psychotomimetic effects in men. While our work demonstrates sex-specific behavioral and neural effects of ketamine, future studies need to continue resolving the sex bias in ketamine research.

## Supplemental Material

sj-docx-1-tpp-10.1177_20451253251406059 – Supplemental material for Sex, drugs, and arousal—two randomized trials on the effects of ketamine on sexual arousal and calcarine gyrus activity Supplemental material, sj-docx-1-tpp-10.1177_20451253251406059 for Sex, drugs, and arousal—two randomized trials on the effects of ketamine on sexual arousal and calcarine gyrus activity by Manfred Klöbl, Thomas Liebe, Gregor Dörl, Peter Stöhrmann, Clemens Schmidt, Elisa Briem, Christian Milz, Gabriel Schlosser, Maximilian Kathofer, David Gomola, Godber Mathis Godbersen, Julia Sophia Crone, Rupert Lanzenberger and Marie Spies in Therapeutic Advances in Psychopharmacology

sj-pdf-2-tpp-10.1177_20451253251406059 – Supplemental material for Sex, drugs, and arousal—two randomized trials on the effects of ketamine on sexual arousal and calcarine gyrus activity Supplemental material, sj-pdf-2-tpp-10.1177_20451253251406059 for Sex, drugs, and arousal—two randomized trials on the effects of ketamine on sexual arousal and calcarine gyrus activity by Manfred Klöbl, Thomas Liebe, Gregor Dörl, Peter Stöhrmann, Clemens Schmidt, Elisa Briem, Christian Milz, Gabriel Schlosser, Maximilian Kathofer, David Gomola, Godber Mathis Godbersen, Julia Sophia Crone, Rupert Lanzenberger and Marie Spies in Therapeutic Advances in Psychopharmacology

## Declarations

**Ethics approval and consent to participate:** Both studies were approved by the ethics committee of the Medical University of Vienna (ethics numbers 2014/2021 and 1572/2021). All procedures were performed in accordance with the Declaration of Helsinki and the good scientific practice guidelines of the Medical University of Vienna. Written informed consent was obtained from all participants.

**Consent for publication:** Not applicable.

**Availability of data and materials:** The imaging data cannot be shared due to legal and administrative restrictions. The novel stimulus dataset compiled for the studies presented here will be shared upon request to the corresponding author.
