# Supplementary material for: Sex, drugs, and arousal—two randomized trials on the effects of ketamine on sexual arousal and calcarine gyrus activity
Source: Ther Adv Psychopharmacol. 2026 Feb 12;16:20451253251406059. doi: 10.1177/20451253251406059 (PMC12901892; doi:10.1177/20451253251406059)
Supplement: sj-docx-1-tpp-10.1177_20451253251406059 – Supplemental material for Sex, drugs, and arousal—two randomized trials on the effects of ketamine on sexual arousal and calcarine gyrus activity [file sj-docx-1-tpp-10.1177_20451253251406059.docx]

Supplementary information for

Sex, drugs, and arousal – Two randomized trials on the effects of ketamine on sexual arousal and calcarine gyrus activity

Manfred Klöbl^1,2^, Thomas Liebe^3^, Gregor Dörl^1,2^, Peter Stöhrmann^1,2^, Clemens Schmidt^1,2^, Elisa Briem^1,2^, Christian Milz^1,2^, Gabriel Schlosser^1,2^, Maximilian Kathofer^4^, David Gomola^1,2^, Godber Mathis Godbersen^1,2^, Julia Sophia Crone^4^, Rupert **Lanzenberger**^1,2,^**^#^**, Marie Spies^1,2^ (**^#^** corresponding author)

^1^ Department of Psychiatry and Psychotherapy, Medical University of Vienna, Vienna, Austria

^2^ Comprehensive Center for Clinical Neurosciences and Mental Health, Medical University of Vienna, Vienna, Austria

^3^ Department of Psychiatry and Psychotherapy, University of Jena, Jena, Germany

^4^ Vienna Cognitive Science Hub, University of Vienna

# Supplementary experimental procedures

We checked the correct application of the study drug via ketamine plasma levels in both studies.

## Study 1 – Reported sexual arousal after intranasal S-ketamine

### Statistical analysis of general arousal

For a complementary analysis of ketamine effects on general arousal (GA) the scores were modeled as follows using a beta distribution with logit link function:

$$GA\sim drug*orient*sex*time+age+habit+order+\left( 1 | subject \right)+(1|stim)$$

## Comparison of Study 1 and Study 2

For comparability purposes, the subjective sexual arousal ratings from both studies were additionally analyzed with the following common model (i.e., without the study-specific corrections for general arousal and the KSOG):

$$SA\sim drug*orient*sex*time+age+habit+order+\left( 1 | subject \right)+(1|image)$$

# Supplementary results

## Study 1 – Reported general arousal after intranasal S-ketamine

Ketamine increased the reported general arousal for men’s rating of gay stimuli only (Table S1, Figure S1). This was in strong contrast to all women’s ratings and men’s ratings for heterosexual and lesbian stimuli.

## Ketamine effects on task activation

The baseline activation for each task condition and subject sex is shown in Figure S2. Qualitatively, men show stronger activation of visual areas and stronger deactivation around the posterior insula. When correlating the subjective sexual arousal rating with the task activation across conditions, female participants showed stronger activation of the anterior cingulate cortex for stimuli rated as more turning on (Figure S3). This correlation was also present but not significant in male participants (potentially due to fewer men in our sample). On the contrary, subjective ratings significantly correlated with brain activation across the lingual and stretching into the calcarine gyrus in male participants. This effect was completely absent in women.

Figure S4 visualizes the extent of the cluster showing an uncorrected drug by participant sex interaction effect in the sexual arousal task. A part of the cluster in the calcarine gyrus displayed a significant interaction of participant sex and stimulus orientation. Detailed analysis of this smaller cluster revealed the drug by participant sex effect at a relaxed significance threshold.

|  | Study 1:  Sub-acute S-ketamine | |
| --- | --- | --- |
|  | Reported general arousal | |
| Effect | Estimate | 95% CI |
| ketamine | -0.01 | [-0.13, 0.12] |
| ketamine x lesbian | -0.04 | [-0.21, 0.14] |
| ketamine x gay | 0.04 | [-0.13, 0.22] |
| ketamine x male | 0.02 | [-0.17, 0.21] |
| ketamine x lesbian x male | -0.06 | [-0.32, 0.19] |
| ketamine x gay x male | **0.36** | **[0.09, 0.62]** |

Table S1: **Ketamine-related effects on reported general arousal with 95% credible intervals (CI).** Female subject sex, heterosexual stimuli and the placebo condition were used as reference categories. Effects with 95% CIs not covering 0 are highlighted in bold.


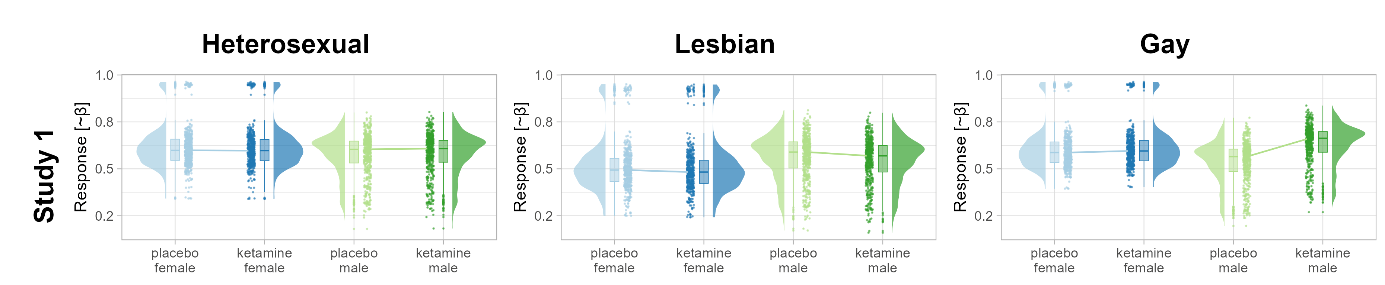


Figure S1: **Fitted reported general arousal per subject and trial.** The values have been adjusted for the model covariates.

## Study 2 – Reported sexual arousal after intravenous racemic ketamine

Table S2 shows the summary statistics for the KSOG separately for each item grouped by participant sex.

|  |  | **f_mean** | **f_std** | **f_median** | **f_IQR** | **f_min** | **f_max** | **m_mean** | **m_std** | **m_median** | **m_IQR** | **m_min** | **m_max** |
| --- | --- | --- | --- | --- | --- | --- | --- | --- | --- | --- | --- | --- | --- |
|  | Sexual attraction - past | 6.05 | 1.39 | 7.00 | 1.00 | 2.00 | 7.00 | 1.56 | 1.50 | 1.00 | 0.50 | 1.00 | 7.00 |
| A | Sexual attraction - present | 5.95 | 1.54 | 6.00 | 1.00 | 1.00 | 7.00 | 1.56 | 1.50 | 1.00 | 0.50 | 1.00 | 7.00 |
|  | Sexual attraction - ideal | 5.63 | 1.74 | 6.00 | 2.75 | 1.00 | 7.00 | 1.69 | 1.49 | 1.00 | 1.00 | 1.00 | 7.00 |
|  | Sexual behavior - past | 6.32 | 1.34 | 7.00 | 1.00 | 2.00 | 7.00 | 1.38 | 1.50 | 1.00 | 0.00 | 1.00 | 7.00 |
| B | Sexual behavior - present | 6.37 | 1.61 | 7.00 | 0.00 | 1.00 | 7.00 | 1.38 | 1.50 | 1.00 | 0.00 | 1.00 | 7.00 |
|  | Sexual behavior - ideal | 5.95 | 1.58 | 7.00 | 1.75 | 1.00 | 7.00 | 1.63 | 1.50 | 1.00 | 1.00 | 1.00 | 7.00 |
|  | Sexual fantasies - past | 5.68 | 1.57 | 6.00 | 2.00 | 1.00 | 7.00 | 1.69 | 1.30 | 1.00 | 1.00 | 1.00 | 6.00 |
| C | Sexual fantasies - present | 5.74 | 1.56 | 6.00 | 1.75 | 1.00 | 7.00 | 1.63 | 1.54 | 1.00 | 0.50 | 1.00 | 7.00 |
|  | Sexual fantasies - ideal | 5.58 | 1.61 | 6.00 | 2.00 | 1.00 | 7.00 | 1.69 | 1.54 | 1.00 | 1.00 | 1.00 | 7.00 |
|  | Emotional preferences - past | 5.21 | 2.04 | 6.00 | 3.00 | 1.00 | 7.00 | 1.63 | 1.63 | 1.00 | 0.00 | 1.00 | 7.00 |
| D | Emotional preferences - present | 5.16 | 1.86 | 6.00 | 3.00 | 1.00 | 7.00 | 1.63 | 1.63 | 1.00 | 0.00 | 1.00 | 7.00 |
|  | Emotional preferences - ideal | 5.32 | 1.67 | 6.00 | 3.00 | 1.00 | 7.00 | 1.63 | 1.63 | 1.00 | 0.00 | 1.00 | 7.00 |
|  | Social preferences - past | 4.32 | 1.42 | 4.00 | 2.00 | 2.00 | 7.00 | 3.75 | 1.53 | 4.00 | 2.50 | 1.00 | 6.00 |
| E | Social preferences - present | 4.32 | 1.49 | 4.00 | 0.75 | 2.00 | 7.00 | 3.63 | 1.36 | 4.00 | 1.00 | 1.00 | 6.00 |
|  | Social preferences - ideal | 4.47 | 1.35 | 4.00 | 0.75 | 2.00 | 7.00 | 3.63 | 1.31 | 4.00 | 0.50 | 1.00 | 6.00 |
|  | Lifestyle preferences - past | 1.95 | 1.27 | 2.00 | 1.00 | 1.00 | 6.00 | 1.56 | 1.36 | 1.00 | 0.00 | 1.00 | 6.00 |
| F | Lifestyle preferences - present | 2.16 | 1.54 | 2.00 | 1.75 | 1.00 | 6.00 | 1.50 | 1.32 | 1.00 | 0.00 | 1.00 | 6.00 |
|  | Lifestyle preferences - ideal | 2.42 | 1.57 | 2.00 | 2.00 | 1.00 | 6.00 | 1.50 | 1.32 | 1.00 | 0.00 | 1.00 | 6.00 |
|  | Self-identification - past | 2.05 | 1.61 | 1.00 | 1.00 | 1.00 | 6.00 | 1.44 | 1.31 | 1.00 | 0.00 | 1.00 | 6.00 |
| G | Self-identification - present | 2.05 | 1.58 | 2.00 | 1.00 | 1.00 | 6.00 | 1.50 | 1.32 | 1.00 | 0.00 | 1.00 | 6.00 |
|  | Self-identification - ideal | 2.16 | 1.61 | 2.00 | 1.75 | 1.00 | 6.00 | 1.50 | 1.32 | 1.00 | 0.00 | 1.00 | 6.00 |

Table S2: **Summary statistics for the Klein Sexual Orientation Grid.** Variables A to E were coded as 1 = “men only” 7 = “women only”. Variables F and G were coded as 1 = “heterosexual only”, 7 = “homosexual only”. f: female, m: male, std: standard deviation, IQR: interquartile range.

| Hetero, female | 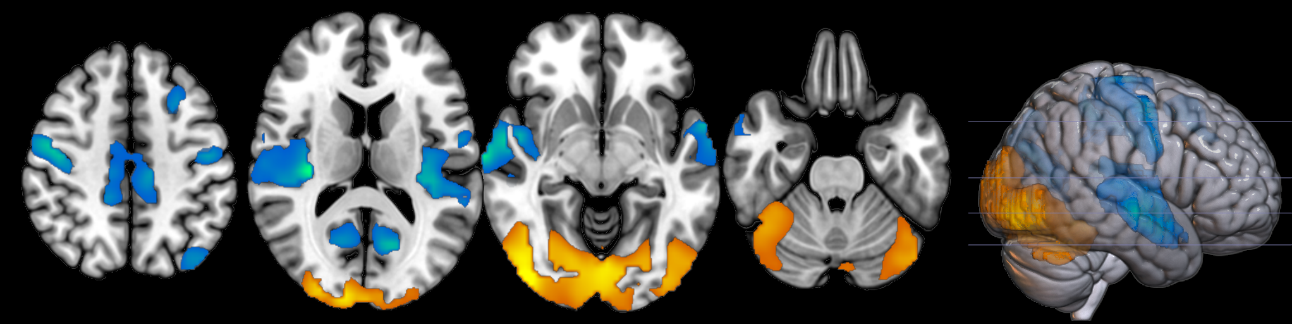 |
| --- | --- |
| Hetero, male | 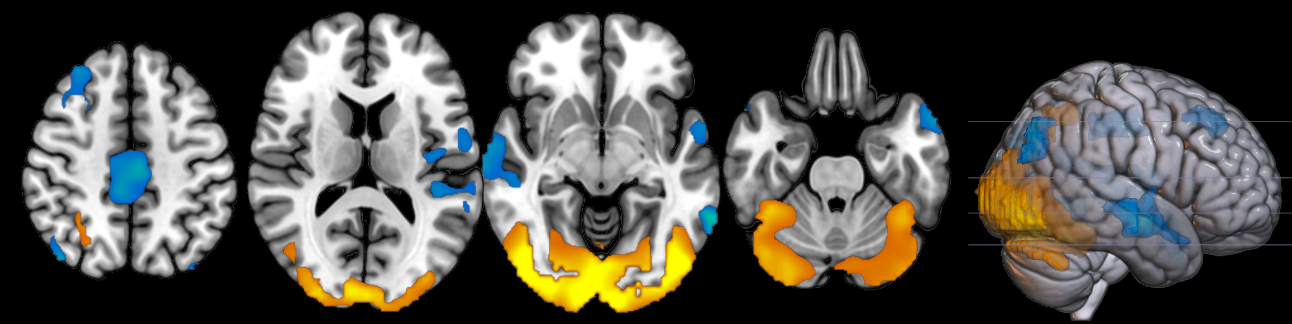 |
| Lesbian, female | 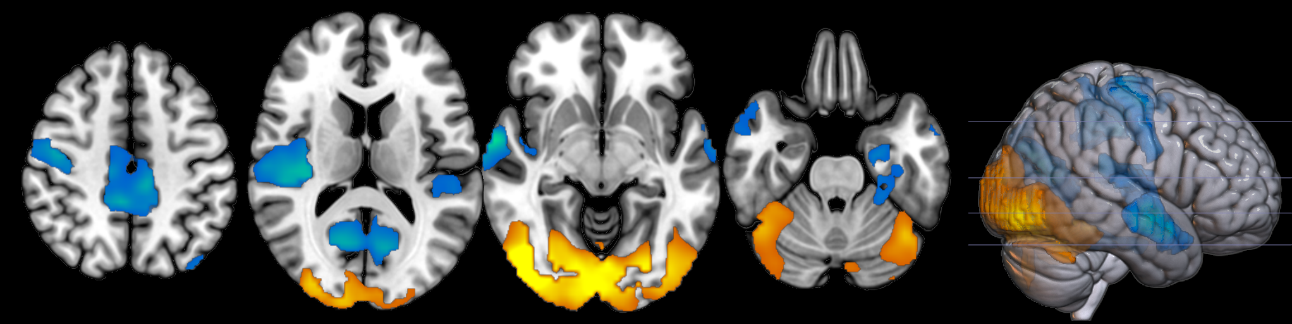 |
| Lesbian, male | 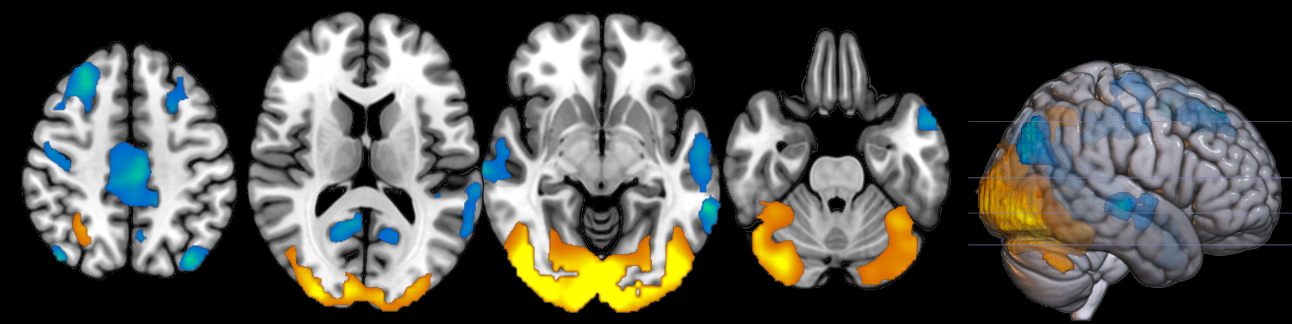 |
| Gay, female | 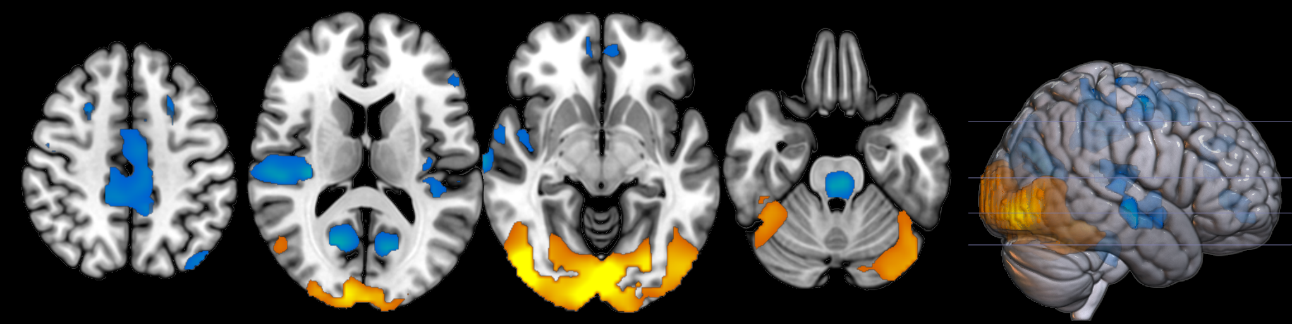 |
| Gay, male | 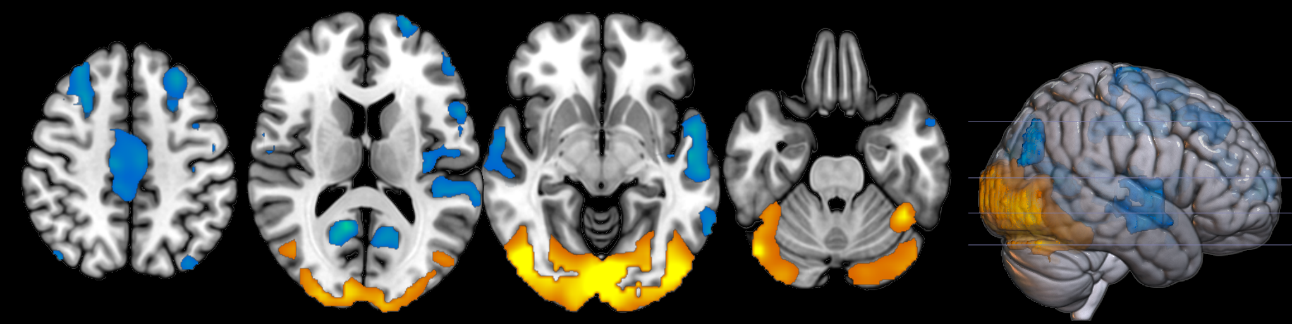 |

Figure S2: **Baseline activation of the sexual arousal task.** Contrasts were calculated for the placebo condition and corrected for age, order of drug application, and the first principal component of the Klein Sexual Orientation Grid (see main text for details). Activations are shown in warm and deactivations in cold colors for clusters significant at a primary threshold of p < 0.001 and familywise error-corrected to p < 0.05 two-sided.

| Female | Male |
| --- | --- |
| 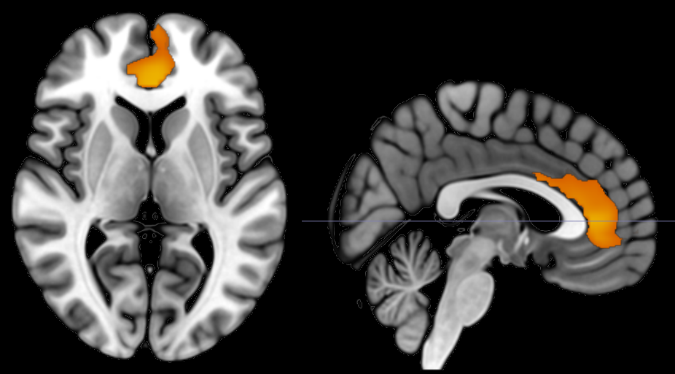 | 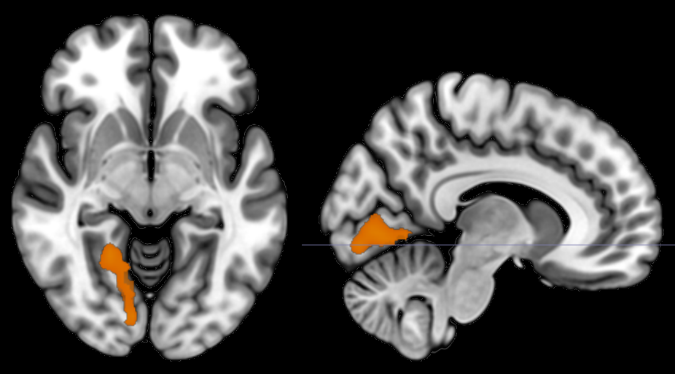 |

Figure S3: **Correlation between task activation and subjective sexual arousal.** Positive correlations are shown in warm colors (no negative correlations survived multiplicity correction) for clusters significant at a primary threshold of p < 0.001 and familywise error-corrected to p < 0.05 two-sided.


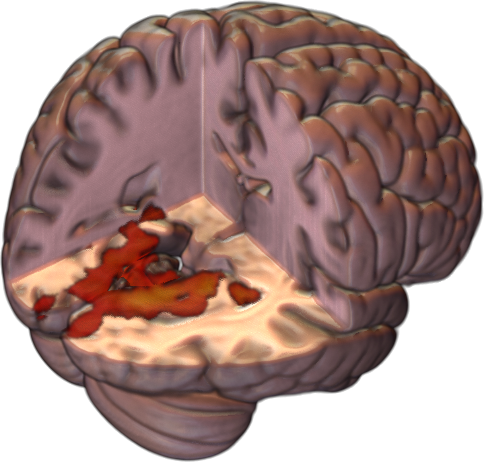


Figure S4: **Extent of the sexual arousal task cluster showing a drug by sex interaction under a relaxed threshold of p < 0.05 uncorrected** (two-sided).

## Comparison of Study 1 and Study 2

Figure S5 shows the data from both studies analyzed with a common model and without study-specific corrections. For Study 1, the most prominent difference are the higher sexual arousal ratings by male participants. Study 2 shows a group of outliers among the male participants with low ratings for all stimuli categories.


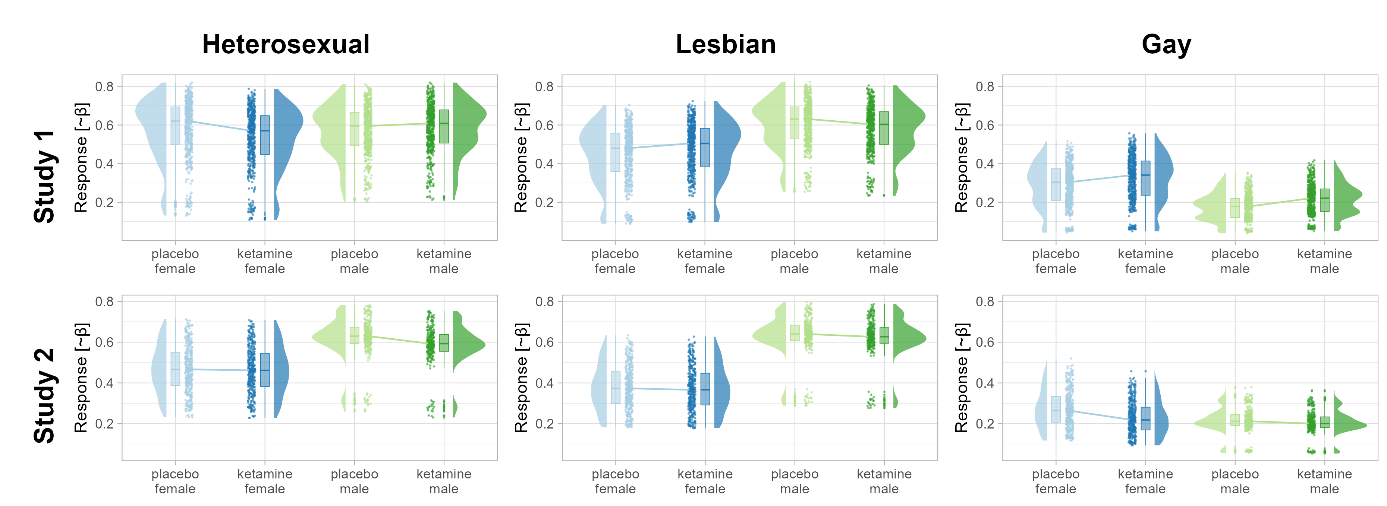


Figure S5: **Fitted reported sexual arousal per subject and trial without study-specific corrections.** The values have been adjusted for the model covariates.

# Supplementary discussion

The increased activation in the anterior cingulate cortex for more sexually arousing stimuli is in line with a previous work differentiating general and sexual arousal (1). However, contrary to our expectations, we did not find any effects in the ventral striatum (1, 2). This discrepancy regarding reward activation could, for instance, be based on the high noise level of the ventral striatum or differences in data preprocessing. The male-specific correlation between subjective sexual arousal and activation in the lingual and calcarine gyrus could be the origin of the observed drug by participant sex and participant sex by stimulus orientation interactions in these regions.

The increased sexual arousal ratings in Study 1 without versus with adjustment for general arousal highlight the overlap between the two domains. If this overlap could lead to biased results or an inability to trace the origin of a potentially observed effect, correction for general arousal is recommended. In particular, the increased subjective sexual arousal in heterosexual men as compared to women for lesbian stimuli might be influenced by an increased general arousal (median and 95% CI for men > women under placebo: 0.70 [0.22, 1.18] without adjustment, 0.33 [-0.21, 0.87] with adjustment). This increased sexual arousal to lesbian stimuli in men has repeatedly been reported in previous works (2-4) despite the popularity of lesbian porn among straight women (5). Of note, a similar overlap between sexual and emotional content is known for brain activation (1).

# Supplementary references

1. Walter M, Bermpohl F, Mouras H, Schiltz K, Tempelmann C, Rotte M, et al. (2008): Distinguishing specific sexual and general emotional effects in fMRI-subcortical and cortical arousal during erotic picture viewing. *Neuroimage*. 40:1482-1494.

2. Klöbl M, Reed MB, Handschuh P, Kaufmann U, Konadu ME, Ritter V, et al. (2024): Gender Dysphoria and Sexual Euphoria: A Bayesian Perspective on the Influence of Gender-Affirming Hormone Therapy on Sexual Arousal. *Arch Sex Behav*. 53:1859-1871.

3. Chivers ML, Rieger G, Latty E, Bailey JM (2004): A sex difference in the specificity of sexual arousal. *Psychol Sci*. 15:736-744.

4. Safron A, Sylva D, Klimaj V, Rosenthal AM, Bailey JM (2020): Neural Responses to Sexual Stimuli in Heterosexual and Homosexual Men and Women: Men's Responses Are More Specific. *Arch Sex Behav*. 49:433-445.

5. Avgar R “Boys just ruin it”: Exploring the popularity of lesbian pornography among straight women. *Sexualities*. 0:13634607241292455.
